# A Brief Review of MicroRNA Profiling in Human Prostate Cancer Tissues and Plasma

**DOI:** 10.3390/biom15081156

**Published:** 2025-08-12

**Authors:** Georgios Kallinikas, Amin M. Ektesabi, Chirag M. Vaswani, Georgios Haronis, Eirini Kallinika, Diomidis Kozyrakis, Evangelos Rodinos, Athanasios Filios, Panagiotis Filios, Despoina Mityliniou, Konstantinos Safioleas, Dimitrios Bozios, Athanasios Karmogiannis, Vasileios Konstantinopoulos, Anna Maria Konomi, James N. Tsoporis

**Affiliations:** 1 Department of Urology, Konstantopouleion–Patision Hospital, N. Ionia, 14233 Attika, Greece; georgioskallinikas@gmail.com (G.K.); george.xarwnhs@gmail.com (G.H.); dkozirakis@yahoo.gr (D.K.); vag.international@hotmail.com (E.R.); athanfilios@gmail.com (A.F.); panosfilios@yahoo.gr (P.F.); dmitiliniou@yahoo.gr (D.M.); konstantinossafioleas@yahoo.gr (K.S.); dbozios@gmail.com (D.B.); krmgti@hotmail.com (A.K.); vkonstantinopoulos@yahoo.com (V.K.); annakonomi24@gmail.com (A.M.K.); 2 Keenan Research Centre for Biomedical Science, Li Ka Shing Knowledge Institute, St. Michael’s Hospital, Unity Health Toronto, Toronto, ON M5B 1W8, Canada; amin.ektesabi@alumni.utoronto.ca (A.M.E.); c.vaswani@utoronto.ca (C.M.V.); 3 Department of Molecular Biology and Genetics, Democritus University of Thrace, 68100 Alexandroupolis, Greece; eirinikallinika@gmail.com

**Keywords:** prostate cancer, biomarkers, tissue biopsies, plasma, microRNA

## Abstract

(1) Background: The gold standard, prostate-specific antigen (PSA) screening lacks the sensitivity and specificity required for confident, early prostate-cancer detection. MicroRNAs (miRNAs) are small, highly stable, non-coding RNAs whose expression changes reproducibly in malignancy and therefore offer promise as minimally invasive biomarkers. Although prostate cancer biopsies are the gold standard for prostate cancer diagnosis, limitations in the field continue to persist. Since circulating fluids can also be a source of miRNA biomarkers, we investigated the overlap between miRNAs enriched in prostate cancer tissue and those isolated from the plasma of patients with prostate cancer. (2) Methods: We synthesized the published literature (PubMed, Google Scholar, ResearchGate, 2005–April 2025) and re-analyzed three Gene Expression Omnibus (GEO) datasets (GSE54516, GSE21032—tissue; GSE206793—plasma) to identify miRNAs consistently dysregulated in prostate cancer tissue and circulation. (3) Results: Of the 318 screened full-text articles, 24 met the inclusion criteria. From the GEO reanalysis (false-discovery-rate < 0.05, |log_2_FC| ≥ 1), 219 and 326 miRNAs were differentially expressed in tissue, whereas 12 were altered in plasma. Two miRNAs—*miR-449b* and *miR-455-3p*—were common in both compartments, highlighting their translational potential as liquid biopsy surrogates of tumor biology. (4) Conclusions: We summarize functional evidence for leading tumor-suppressive (e.g., *miR-205*, *miR-23b*, miR-455-3p) and oncogenic (e.g., *miR-21*, *miR-182*, *miR-449b*) candidates, discuss their intersection with the androgen-receptor, TGF-β, WNT/β-catenin, and PI3K-AKT signaling, and outline outstanding requirements for the clinical qualification of miRNA panels in prostate cancer.

## 1. Introduction

Prostate cancer is recognized as the most diagnosed cancer among men worldwide, the second leading cause of cancer among men in the United States, and the third in Europe [1,2]. In 2020, an estimated 375,000 deaths were attributed to prostate cancer globally, and one in eight men are expected to be diagnosed with prostate cancer in their lifetime. The prevalence of prostate cancer ranges from less than 5% in men under 30 years of age to nearly 60% in men over 79. Despite its frequency, most men diagnosed with prostate cancer do not die from it. In the United States, the majority of the 3,300,000 men who have received a diagnosis remain alive. Between 1993 and 2013, a nearly 50% reduction in prostate cancer mortality was observed, primarily attributed to earlier diagnosis. The estimated mean cost for prostate cancer–specific treatment is USD 2935, including USD 2537 before, and USD 6661, after progression to castration-resistant prostate cancer [3]. The etiology of prostate cancer remains largely unknown, although ethnic background and family history are associated with increased incidence, suggesting a genetic implication.

MicroRNAs (miRNAs) have recently garnered significant attention, as the 2024 Nobel Prize in Physiology or Medicine was awarded to Victor Ambros and Gary Ruvkun “for the discovery of microRNA and its role in post-transcriptional gene regulation” [4]. A microRNA (miR or miRNA) is a small, non-coding RNA molecule, about 20–25 nucleotides long, that plays a key role in regulating gene expression. Instead of coding for proteins, miRs bind to messenger RNAs (mRNAs), typically at the 3′ untranslated region (3′ UTR), and either block the translation of the mRNA into protein or cause the mRNA to be degraded [5]. This means miRs act like molecular switches, helping to fine-tune which proteins get made and in what amounts. miRs have been linked to developmental, physiological, and behavioral defects in animals [5]. miRNAs occur in both animals and humans [6].

miRNAs are initially transcribed in the nucleus by Polymerase II as “pri-miRNAs,” which possess at least one hairpin region. With the involvement of the Drosha endonuclease and DGCR8 protein, the hairpin loop of pri-miRNA is cleaved to produce “pre-miRNA” [7]. Exportin 5 facilitates the transfer of pre-miRNA to the cytoplasm, where Dicer cleaves both strands near the loop, generating the miRNA–miRNA* duplex (the miRNA and its passenger strand). This duplex is then loaded onto an Argonaute protein. An ATP-dependent process releases the mature miRNA from Argonaute, while the passenger strand is removed. The resulting mature miRNA is the active form that binds to mRNA and mediates post-transcriptional repression.

In cancer, the miR regulatory function becomes especially important. Some miRs act as tumor suppressors, helping prevent uncontrolled cell growth by silencing genes that drive proliferation. Others act as oncomiRs, promoting cancer development by blocking tumor-suppressing genes. For example, if a tumor-suppressive miR is lost or downregulated, cancer cells may grow unchecked. Conversely, if an oncomiR is overproduced, it can silence key brakes on cell division. Because of this dual nature, miRs are being studied as both biomarkers (prognostic/diagnostic) and potential therapeutic targets in many types of cancer [8,9].

The principal goal of this review article is to provide an overview of current knowledge regarding the impact of miRNAs on prostate cancer and their potential applications in the future.

## 2. Methods

### 2.1. Systemic Review

A systematic literature review was conducted in PubMed, Google Scholar, and ResearchGate using the terms “prostate cancer,” “prostate cancer diagnosis,” “prostate cancer treatment,” and “miRNA,” with no time restrictions. This search returned 318 articles, of which 310 were excluded for failing to meet the selection criteria. An additional 16 articles were identified through reference lists. A full-text assessment was performed for all selected articles, and 24 were finally included.

### 2.2. Inclusion Criteria

- Studies focusing on the impact of miRNAs on prostate cancer.
- Peer-reviewed articles.
- Reviews (any type), meta-analyses, clinical trials.
- Research regarding miRNAs as a diagnostic or therapeutic tool in prostate cancer.

### 2.3. Exclusion Criteria

- Publications focusing on cancer types other than prostate cancer.
- Publications not in the English language.
- Lack of full-text availability.

Three team members independently assessed each selected study for research type, sample size, results, and conclusions. Potential limitations, such as small sample size or bias, were acknowledged, and recommendations for further investigation with larger sample sizes were made.

## 3. Results and Discussion

### 3.1. miRNAs-Based Biomarkers

Across 24 eligible studies and 2 public GEO tissue datasets, >150 miRNAs show reproducible dysregulation in prostate cancer tissue.

Like other malignancies, prostate cancer presents a distinctive miRNA expression profile [10,11,12]. An early microarray study by Porkka et al. [13] already captured this breadth, reporting 37 tumor-suppressive losses and 14 oncogenic gains relative to normal prostate. Since then, functional screens, such as the CRISPR/Cas9 knockout series, by using a pooled CRISPR/Cas9 knockout screen, Jiang et al. [14] confirmed that loss of tumor-suppressive *miR-205*, *miR-23b*, and *miR-30c* accelerates LNCaP-cell proliferation, whereas the enforced expression of the oncogenic pair *miR-1225-5p*/*miR-663a* has the opposite effect. Full gene lists, fold-changes, and *p* values are summarized in Table 1. A lentiviral vector knockout system was used on LNCaP cells to study these miRNAs. Significant enhancement (*p* < 0.05) was observed in cell proliferation following knockout of *miR-222*, *miR-224*, *miR-23b*, *miR-205*, and *miR-30c*, whereas knockout of *miR-1225-5p* and *miR-663a* caused a notable reduction in proliferative potential (*p* < 0.01). Knockout of *miR-221*, *miR-455-3p*, and *miR-505* did not show any significant changes (*p* > 0.05) in proliferation. Regarding cell invasion, knockout of *miR-205*, *miR-221*, *miR-455-3p*, *miR-222*, *miR-224*, *miR-505*, *miR-23b*, and *miR-30c* contributed to increased invasiveness in LNCaP cells, suggesting possible tumor-suppressive functions [14]. Several studies [15,16,17] emphasized *miR-205* as a tumor suppressor in prostate cancer, and Verdoot et al. [18] demonstrated its role in DNA damage response following doxorubicin or cisplatin treatment, as well as in inhibiting proliferation in PC-3 and LNCaP cells.

Numerous studies [20,24,30,31] supported the notion that *miR-221* and *miR-222* are downregulated in prostate cancer, and additional findings suggested that the loss of miR-30c may enhance the proliferation and invasiveness of cancer cells [20]. Jiang et al. [14] first proposed that *miR-663a* and *miR-1225-5p* act as tumor promoters. Zhang et al. [21] reported that *miR-335-5p* can induce apoptosis in prostate cancer cells and proposed it as a potential biomarker [21,32]. Sun et al. [23] found that aggressive prostate cancers tend to overexpress oncogenic *miR-21*, *miR-125b*, *miR-221*, and *miR-222*. Nikitina et al. [22] associated overexpressed *miR-21* with tumor growth, possibly by knocking down PTEN and other tumor-suppressor genes [2,22]). Feng et al. [33] examined the miR-200 family and noted its key role in suppressing tumor-suppressive mechanisms and regulating cell migration. Siegel et al. [34] further observed that combining these five miRNAs with routine PSA tests may improve diagnostic accuracy.

Shinawi T. et al. [35] identified 53 dysregulated miRNAs, and 250 target genes involved in Hedgehog, ErbB, and cAMP signaling pathways in prostate cancer metastasis. Their study narrowed the hub miRNAs to *hsa-miR-455-3p*, *hsa-miR-548c-3p*, and *hsa-miR-582-5p*, and the hub genes to *NFIB*, *DICER1*, *GSK3B*, *DCAF7*, *FGFR1OP*, *ABHD2*, *NACC2*, *NR3C1*, and *FGF2*. *NR3C1*, *ABHD2*, and *GSK3B* displayed notable mutation capacity and altered expression. The downregulation of *NR3C1* was linked to patient survival beyond 150 months. *Hsa-miR-582-5p* targeted the *NR3C1*, *ABHD2*, and *GSK3B* genes and appeared to correlate with the increased expression of *ABHD2* and *GSK3B* in prostate tissues. Earlier work by Gordanpour et al. [36] and Huang et al. [37] suggested that *hsa-miR-582-5p* might inhibit metastasis by repressing TGF-β signaling. Feng et al. [38] examined the role of *miR-548c-3p* as a cancer regulator, particularly through ErbB and Hippo pathways.

Wong A. et al. [39] explored clinical and molecular markers of long-term survival following oligometastasis-directed stereotactic body radiotherapy and highlighted that miRNAs can regulate tumor behavior. *MiR-23b*, which targets oncogenes and signaling molecules (*PTEN*, *Akt*, *SRC*, *MAP3K1*, *TGFbR2*, *RRAS2*), was shown to inhibit proliferation, migration, invasion, epithelial–mesenchymal transition, angiogenesis, and metastasis in animal models [40,41,42,43]. Clinical studies [40,41,42,43,44,45] reported improved overall survival in patients with various malignancies, including prostate cancer, when *miR-23b* was upregulated. *MiR-449a* and *miR-449b* also share multiple overlapping targets, such as *CDK6*, *CDC25A*, *HDAC1*, *MET*, and *FOS* [45,46,47,48]. In prostate cancer, elevated *miR-449b* is associated with increased recurrence risk [27], although both *miR-449a* and *miR-449b* have shown tumor-suppressive effects in vitro [45,46,47,48]. These findings highlight the complexity of miRNA pathways in prostate cancer pathogenesis.

### 3.2. miRNAs in Locally Advanced Prostate Cancer

Pudova et al. [25] compared node-positive (N1, *n* = 20) with node-negative (N0, *n* = 24) tumors and reported 18 differentially expressed miRNAs. Eight belonged to two oncogenic clusters—*miR-183-96-182* and *miR-25-93-106b*—already implicated in epithelial-to-mesenchymal transition (EMT) and early dissemination. Conversely, the N0 group was enriched for tumor-suppressive species such as *miR-143-5p* and *miR-455-3p*, both linked to TGF-β antagonism. Table 2 summarizes the nine N1-enriched miRNAs, their predicted targets (*PTEN*, *E-cadherin*, *FOXO1*), and supporting references. The dominance of the *miR-183-96-182* cluster suggests cooperative regulation of metastatic competence and pinpoints this tri-miRNA cassette as a potential liquid biopsy marker for lymph node involvement.

#### 3.2.1. N1 Group

Among the nine miRNAs in the N1 group, *miR-182-5p*, *miR-183-5p*, and *miR-96-5p* belong to the *miR-183-96-182* oncogenic cluster, which has been widely studied for its oncogenic properties. *miR-183* contributes to tumor invasion and metastasis by targeting *PDCD4*, *PP2A*, *EGR1*, and *PTEN* [38,39,40,]. These three miRNAs also appear to negatively affect *FOXO3a*, a tumor suppressor gene responsible for cell cycle arrest and cell death in endometrial cancer [52]. Some findings suggest that *FOXO1* may act as a repressor of the androgen receptor, which is a principal oncogenic pathway in prostate cancer [53]. Siu MK et al., 2015, reported that elevated *miR-96-5p* in prostate cancer promotes metastasis by activating the mTOR pathway [26]. Similarly, Schaefer et al. [24], Casanova-Salas [54], and Wang et al. [55] observed increased *miR-182-5p* in prostate cancer tissues, differentiating them from normal tissues with nearly 100% specificity.

Two other miRNAs, *miR-25* and *miR-93*, were also elevated in the N1 samples. These, together with *miR-106*, form the *miR-106b-25* oncogenic cluster, which is elevated in multiple malignancies including gastric, prostatic, pancreatic neuroendocrine cancers, multiple myelomas, and neuroblastomas. This cluster, alongside the *miR-17-92* oncogenic cluster, regulates *TGF-β*. Its inactivation is considered a key step in tumor progression because it disrupts cell apoptosis and cell cycle arrest [56]. *miR-25* has been reported to be elevated in several cancers, including prostate cancer [57], and *miR-25-3p* levels have been specifically associated with prostate cancer [58,59]. Choi et al. [17] reported a correlation between the progression of cancer and increased *miR-93-5p*. Another oncogenic miRNA, *miR-615-3p*, is elevated in many cancers, including aggressive prostate cancer [57,58,59].

#### 3.2.2. N0 Group

Although miRNAs often act as suppressors, their roles can be ambivalent and depend on specific biological contexts. *miR-221* and *miR-222* are decreased in the N1 group. Galardi et al. [19] found that these miRNAs target the cell cycle inhibitor p27, thereby influencing cancer cell proliferation. They normally regulate vascular remodeling after injury by controlling endothelial cell differentiation, migration, and proliferation [60,61]. Numerous studies have investigated these miRNAs in various cancers, including prostate cancer, where they may function as either tumor suppressors or oncogenes [19,60,61,62,63,64]. Kiener et al. [65] employed mouse and zebrafish models to investigate whether *miR-221-5p* controls proliferation and migration in human prostate cancer cells and observed a reduction in *miR-221-3p* and *miR-221-5p* during prostate cancer progression. Moreover, *miR-221-5p* appears to function as a tumor suppressor in prostate cancer cell lines, limiting tumor burden. *miR-221* serves as a key regulator of a network of other miRNAs in prostate cancer and can influence cell physiology [66]. Thus, the exact biological roles and mechanisms of action of *miR-221* and *miR-222* in androgen-independent prostate cancer pathogenesis are not yet fully understood.

Multiple reports describe *miR-223-5p* as an abnormally expressed lagging strand in various solid malignant tumors, including vulvar cancer [67], non-small cell lung cancer [68], and bladder cancer [69]. Evidence suggests that *miR-223-5p* acts as an anti-tumor miRNA by targeting oncogenic genes such as *E2F8* and *ALN*. The ETS-related gene (*ERG*), a member of the ETS family, is crucial in hematopoiesis and angiogenesis [70]. In approximately half of prostate cancer patients, the *TMPRSS2-ERG* fusion gene occurs [71], which may upregulate *ERG* and promote cell development [72]. *ERG* is hypothesized to drive epithelial neoplasia and cancer progression in prostate epithelium.

Wei et al. [28] investigated the correlation between *miR-223-5p* and *ERG*. In multiple prostate cancer cell lines, an inverse relationship was identified between *miR-223-5p* and *ERG* expression. Potential targets of *miR-223-5p* were then predicted using Targetscan, miRDB, microRNA, and PhastCons, indicating *ERG* as a likely target. DU145 cells were transfected with *miR-223-5p* mimics (miR-MM) or *miR-223-5p* antisense oligonucleotides (miR-AO). A downregulation of *ERG* was observed in miR-MM cells, while an upregulation was detected in miR-AO cells, thus supporting the negative regulation of *ERG* by *miR-223-5p*. Further experiments in DU145 and LNCaP cells, each transfected with one of three *ERG* siRNAs (to select the optimal one), demonstrated that silencing *miR-223-5p* resulted in a marked increase in *ERG* expression, suggesting a negative feedback loop. Knocking down *ERG* significantly inhibited cell proliferation, migration, and invasion in prostate cancer cell lines, emphasizing the potential oncogenic function of *ERG* and its regulation by *miR-223-5p*.

#### 3.2.3. Androgen Receptors and miRs

Androgen receptors play an important role in the normal growth of prostate cells. Their regulatory actions and mechanisms remain incompletely understood in prostate cancer. Ostling et al. [50] investigated several miRNAs that either upregulate or downregulate androgen receptor protein levels by targeting the 3′UTR. They reverse-transfected 21 miRNAs into LNCaP and 22Rv1 cell lines and performed Western blot and qRT-PCR to analyze androgen receptor levels, finding that these miRNAs reduced the androgen receptor protein. Most also decreased PSA levels in LNCaP cells, although *miR-299-3p* increased PSA. *miR-30d* increased both PSA and androgen receptors in LNCaP cells, while *miR-9* decreased PSA levels. The 22Rv1 cells can express an isoform of the androgen receptor [73,74], and several miRNAs—*miR-135b*, *miR-147*, *miR-299-3p*, *miR-34a*, and *miR-644*—downregulated this alternative transcript. In addition, *miR-147*, *miR-297*, *miR-298*, *miR-299–3p*, *miR-421*, and *miR-449a* reduced the androgen receptor mRNA in both LNCaP and 22Rv1 cells. *miR-371–3p*, *miR-449b*, and *miR-491–5p* were particularly effective in LNCaP cells, while *miR-876–3p* was more effective in 22Rv1. Out of the 21 miRNAs, 13 (*miR-135b*, *miR-185*, *miR-297*, *miR-299–3p*, *miR-34a*, *miR-34c*, *miR-371–3p*, *miR-421*, *miR-449a*, *miR-449b*, *miR-634*, *miR-654–5p*, and *miR-9*) interacted with the 3′UTR region of the androgen receptor. Among these, *miR-421*, *miR-449a*, *miR-449b*, and *miR-9* also reduced exogenous androgen receptor expression [73,74].

*miR-34a* and *miR-34c* are regulated by the tumor suppressor TP53, meaning that decreased levels of these miRNAs can lead to increased tumor aggression, elevated PSA, and metastasis [51]. In 47 prostate tumors, *miR-34a* and *miR-34c* levels and androgen receptor content were measured by qRT-PCR and immunostaining, respectively. An inverse correlation was found between *miR-34c* levels and androgen receptor immunostaining (*p* = 0.0082), and similarly for *miR-34a* (*p* = 0.0085). Both miRNAs directly target the 3′UTR region of the androgen receptor, influencing prostate cancer progression.

### 3.3. PSA vs. Circulating miRNAs in Prostate Cancer

Although the prostate biopsy is the gold standard prostate cancer diagnostic tool, and the identification of miRs in a prostate biopsy has been the focus of intense research, as outlined in Table 1 and Table 2, it is bound by several limitations [75]. Most prostate biopsies are routinely performed by taking 12 cores under the transrectal ultrasound guidance [75], with an increase in the core number found to increase the cancer detection rate by only 1.06-fold [76]. However, the major drawback lies in the possibility of generating false negatives, as the samples are often taken randomly due to the unknown location of the tumor, and patients may require repeated biopsies under MRI guidance or in combination with ultrasound for better sensitivity [75]. Non-invasive quantification of circulating miRNAs in body fluids, specifically plasma, may serve as clinically important biomarkers of prostate cancer.

### 3.4. Computational Analysis

**Figure 1:**
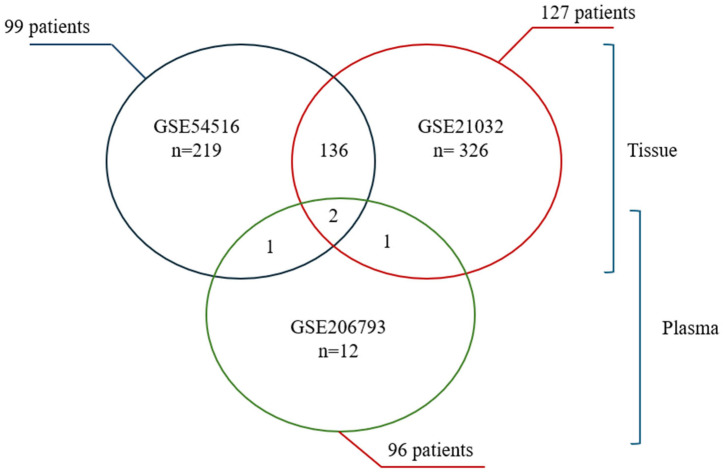
Venn diagram of differentially expressed miRNAs in prostate cancer. This schematic depicts the overlap of significantly dysregulated miRNAs from three GEO datasets: GSE54516 (219 miRNAs, 99 patients, tissue), GSE21032 (326 miRNAs, 127 patients, tissue), and GSE206793 (12 miRNAs, 96 patients, plasma). Numbers in the overlapping and non-overlapping regions illustrate the count of miRNAs unique to or shared among each dataset. Notably, *miR-449b* and *miR-455-3p* were common to tissue and plasma, highlighting their potential value for early prostate cancer detection and monitoring.

In this study, three publicly available prostate cancer (PC) miRNA datasets from the Gene Expression Omnibus (GEO) were selected to identify and compare differentially expressed miRNAs in both tissue and plasma. GSE54516 (GEO set 1, 219 miRNAs, 99 patients) [77] and GSE21032 (GEO set 2, 326 miRNAs, 127 patients) [78] focused on PC tissue, whereas GSE206793 (GEO set 3, 12 miRNAs, 96 patients) [79] examined miRNAs from plasma samples. All datasets were reviewed for quality, completeness, and normalization parameters prior to analysis. Differential expression testing was performed with Transcriptome Analysis Console (TAC) software, applying *p* < 0.05 and a false discovery rate (FDR) < 0.05. In GSE206793, multiple binary comparisons were performed between the four available cohorts (healthy volunteers, low risk, intermediate risk, and high risk). The only comparison that yielded a statistically significant set of differentially expressed miRNAs (FDR < 0.05, |log_2_FC| ≥ 1) was between healthy controls (*n* = 5) and the high-risk prostate cancer group (*n* = 91). As a result, this comparison was used for downstream analysis and is reflected in the Venn diagram (Figure 1). Comparisons involving the low- and intermediate-risk groups did not meet the criteria for differential expression and were therefore excluded. For GSE54516 and GSE21032, the comparisons were straightforward as they involved prostate cancer patients and healthy/benign controls only. In both cases, the entire dataset was used to identify significantly dysregulated miRNAs using the same statistical thresholds.

As shown in Figure 1, *miR-449b* and *miR-455-3p*, discussed below, were identified in both tissue and plasma signatures, suggesting they may serve as circulating biomarkers for prostate cancer. These two miRNAs may be associated with the primary tumor site and could be indicative of prostate cancer at an early stage.

#### 3.4.1. *miR-455-3p*

Cap-dependent translation is recognized as an important process in the initiation and progression of many cancers, partially through the translation of oncogenic mRNAs such as *cyclin D1* and *c-MYC* [80]. The *eIF4F* complex regulates cap-dependent translation [81]. Zhao et al. [29] investigated the role of *miR-455-3p* in prostate cancer and noted that it targets and suppresses *eIF4E*. Using quantitative PCR, they assessed *miR-455-3p* in one benign cell line and five cancer cell lines (22Rv1, LNCaP, C4-2, PC-3, and DU145), detecting a significant reduction in *miR-455-3p* in cancer cells relative to benign cells. Analysis of 65 clinical prostate tissue samples (18 benign, 47 cancerous) showed a marked decrease in *miR-455-3p* levels in cancer tissues compared to normal tissues, suggesting a role in cancer initiation. Conversely, *miR-455-3p* upregulation appeared to reduce cancer cell growth. When a *miR-455-3p* mimic was introduced into LNCaP and PC-3 cells, their proliferation declined in vitro; the in vivo overexpression of *miR-455-3p* in PC-3 xenografts also suppressed tumor growth.

To confirm the inhibitory function of *miR-455-3p*, an antagomir was used to silence endogenous *miR-455-3p*, leading to enhanced cancer cell proliferation in vitro and in vivo. Further experiments indicated that *miR-455-3p* inhibits cap-dependent translation and prostate cancer cell proliferation via its effect on *eIF4E*. The introduction of a 3′ UTR–deleted *eIF4E* plasmid into *miR-455-3p*–transfected PC-3 cells reversed the inhibition of cap-dependent translation and cell growth. However, upon silencing *eIF4E*, the observed increase in cell activity was attenuated, suggesting that the interplay between *miR-455-3p* and *eIF4E* underlies its regulatory effects on prostate cancer cell proliferation.

#### 3.4.2. *miR 449b*

Three independent cohorts agree that *miR-449b* is the strongest predictor of biochemical recurrence (BCR) after radical prostatectomy. In Mortensen et al. [27], a 2.8-fold increase translated to a hazard ratio (HR) of 1.9 (*p* = 0.003). Fendler et al. [82] later validated *miR-449b* (late BCR) alongside *miR-10b* (early BCR), while Prueitt et al. [83] highlighted *miR-126* and *miR-125-5p* as protective. Ambs et al. [10] highlighted the role of elevated *miR-449b* and *miR-484* in extra prostatic disease extension.

Some findings indicate that *miR-449b* can inhibit androgen receptor expression, which in turn suppresses androgen-driven cell proliferation [84]. Noonan et al. [85] further reported that the E2F1 transcription factor activates the transcription of the *miR-449* cluster, leading to cell cycle arrest and apoptosis through the inhibition of CDK6 and CDC25A—indicative of a negative feedback mechanism against E2F1-mediated cell proliferation. This tumor-suppressive capacity of the *miR-449* cluster has been observed in multiple cell lines (prostate, breast, and lung) via different mechanisms [85,86]. Additional interactions with *LEF-1*, a known effector of the WNT pathway, have also been proposed [87]. The *miR-449* cluster may limit WNT signaling by inhibiting *LEF-1* [88,89], which can reduce cancer growth, including prostate cancer cells. Furthermore, *miR-449* targets the NOTCH pathway and alters cell differentiation [90]. As the NOTCH pathway promotes both epithelial-to-mesenchymal transition (EMT) and cancer progression—and is linked to reduced bone formation—*LEF-1* inhibition by *miR-449b* could potentially promote bone metastasis in prostate cancer. Inhibiting *LEF-1* can decrease osteoblast differentiation and bone density, potentially facilitating metastasis of prostate cancer cells to the skeleton [91].

## 4. Conclusions and Limitations

This review should be interpreted in consideration of several important limitations. Firstly, most studies relied on small cohorts, where many tissue and nearly all plasma studies included 60 patients or fewer. This constrains statistical power, especially for miRNAs present at low abundance, and makes it hard to analyze subgroups by stage or ancestry. Secondly, pre-analytical procedures varied widely. Some groups collected plasma in EDTA tubes, and others used serum; the time to process and extract kits also differed. Such differences can change hemolysis levels and miRNA yield by more than twofold, so direct comparisons must be interpreted with caution. Thirdly, discovery platforms were not uniform. Microarrays, small-RNA sequencing, and fixed qPCR panels each have distinct dynamic ranges and background correction methods, meaning that a miRNA “missing” in one dataset may be below that platform’s detection limit. And finally, there was limited external replication beyond *miR-21*, *miR-449b*, and *miR-455-3p*. These few candidates have been validated in three or more independent cohorts. Until these issues are addressed, any proposed biomarker panel should be considered exploratory.

**Figure 2:**
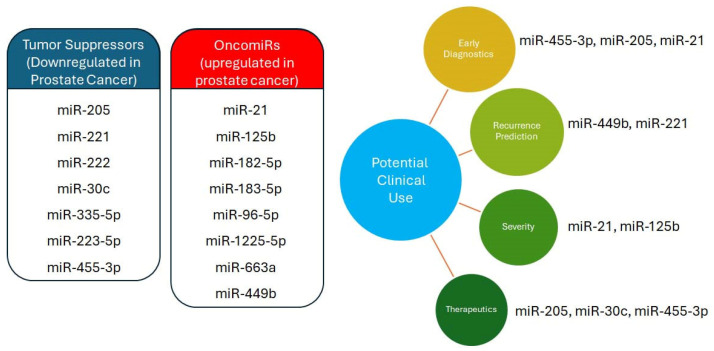
Schematic summarizing miRs regulated in prostate cancer and their potential clinical use.

Nonetheless, by examining the complex roles of various miRNAs in prostate cancer progression, this study aimed to identify their potential roles as prognostic markers and therapeutic targets, as summarized in Figure 2. Restoring the levels of *miR-205*, *miR-30c*, *miR-23b*, *miR-455-3p*, and *miR-222*—often found downregulated in prostate cancer—may help control cell proliferation, migration, and invasion. Conversely, inhibiting *miR-21*, *miR-1225-5p*, *miR-663a*, *miR-449b*, *miR-182-5p*, *miR-183-5p*, *miR-96-5p*, *miR-25*, *miR-93*, and *miR-615-3p* could impede disease progression. In advanced cases, *miR-582-5p* and *miR-548c-3p*, which intersect with pathways such as TGF-β, Hedgehog, and ErbB, may confer additional benefits.

The involvement of *miR-34a*, *miR-34c*, and miR-449b in androgen receptor regulation and pathways such as WNT, NOTCH, and TGF-β may also provide future therapeutic options. *miR-141*, *miR-335-5p*, and *miR-449b*, which correlate with patient recurrence and survival, hold promise as prognostic markers. Particularly, *miR-449b* is linked to a higher risk of biochemical recurrence after radical prostatectomy, while *miR-141* is notably stable in blood samples. Furthermore, incorporating *miR-455-3p*, *miR-23b*, *miR-200* family members, and *miR-221/222* with traditional PSA testing has shown improved diagnostic sensitivity and specificity, aiding in earlier detection and enhanced risk stratification.

miRNAs offer a mechanistically informative and clinically accessible window into prostate-tumor biology. Integrative tissue-and-plasma analysis identifies *miR-449b* and *miR-455-3p* as robust, concordant biomarkers with plausible functional relevance. Standardized pipelines and large-scale trials will be essential to translate these insights into routine urologic practice.

## Figures and Tables

**Table 1:** miRNAs as predictive biomarkers in prostate cancer.

miRNA	Expression in Prostate Cancer	Location (Plasma/Tissue/Both)	Predictive Role	Authors
*miR-205*	Downregulated	Tissue	Tumor suppressor; predictive of cancer progression	[13,14]
*miR-221*	Downregulated	Tissue	Predictive of tumor suppression and cancer progression	[14,19]
*miR-222*	Downregulated	Tissue	Predictive of tumor suppression and cancer progression	[14,19]
*miR-30c*	Downregulated	Tissue	Predictive of cancer cell proliferation and invasion	[14,20]
*miR-1225-5p*	Upregulated	Tissue	Predictive of tumor promotion	[14]
*miR-663a*	Upregulated	Tissue	Predictive of tumor promotion	[14]
*miR-335-5p*	Downregulated	Tissue	Predictive of apoptosis induction in prostate cancer	[21]
*miR-21*	Upregulated	Tissue	Predictive of aggressive prostate cancer	[15,22]
*miR-125b*	Upregulated	Tissue	Predictive of aggressive prostate cancer	[23]
*miR-182-5p*	Upregulated	Tissue	Predictive tumor growth and differentiation	[24,25]
*miR-183-5p*	Upregulated	Tissue	Predictive of tumor invasion and metastasis	[25]
*miR-96-5p*	Upregulated	Tissue	Predictive of metastatic potential	[25,26]
*miR-449b*	Upregulated	Tissue	Predictive of biochemical recurrence after radical prostatectomy	[27]
*miR-223-5p*	Downregulated	Tissue	Predictive of tumor suppression via ERG inhibition	[28]
*miR-455-3p*	Downregulated	Tissue	Predictive of tumor suppression via eIF4E inhibition	[29]

**Table 2:** miRNAs as therapeutic targets in prostate cancer.

miRNA	Expression in Prostate Cancer	Location (Plasma/Tissue/Both)	Therapeutic Role	Authors
*miR-205*	Downregulated	Tissue	Tumor suppressor; potential therapeutic target for restoring tumor suppression	[15,32]
*miR-221*	Downregulated	Tissue	Therapeutic target for inhibiting tumor progression	[14,19]
*miR-222*	Downregulated	Tissue	Therapeutic target for inhibiting tumor progression	[14,19]
*miR-30c*	Downregulated	Tissue	Therapeutic target for reducing cancer cell proliferation and invasion	[14,20]
*miR-1225-5p*	Upregulated	Tissue	Therapeutic target for inhibiting tumor promotion	[14]
*miR-663a*	Upregulated	Tissue	Therapeutic target for inhibiting tumor promotion	[14]
*miR-21*	Upregulated	Tissue	Therapeutic target for reducing tumor growth and PTEN knockdown	[22,49]
*miR-200* family	Downregulated	Tissue	Therapeutic target for regulating cancer cell migration and tumor suppression	[33]
*miR-23b*	Downregulated	Tissue	Therapeutic target for inhibiting tumor proliferation, migration, and metastasis	[40,42]
*miR-449a/b*	Downregulated	Tissue	Therapeutic target for inducing cell cycle arrest and apoptosis	[47,48]
*miR-582-5p*	Downregulated	Tissue	Therapeutic target for inhibiting TGF-β signaling and metastasis	[35,37]
*miR-548c-3p*	Downregulated	Tissue	Therapeutic target for regulating ErbB and Hippo signaling pathways	[38]
*miR-455-3p*	Downregulated	Tissue	Therapeutic target for inhibiting eIF4E and tumor growth	[29]
*miR-223-5p*	Downregulated	Tissue	Therapeutic target for inhibiting ERG and tumor progression	[28]
*miR-34a/c*	Downregulated	Tissue	Therapeutic target for reducing androgen receptor levels and tumor aggression	[50,51]

## Data Availability

All data are presented in the manuscript.
